# Design, protocol and baseline data of Nurturing Healthy Teachers, a cluster non-randomized controlled trial to improve the health, well-being, and food security of preschool and elementary school teachers in Houston, Texas

**DOI:** 10.1016/j.pmedr.2024.102674

**Published:** 2024-03-01

**Authors:** Shreela V. Sharma, Mackenzie Senn, Angela Zieba, Miao Tang, Ru-Jye Chuang, Courtney Byrd-Williams, Mike Pomeroy, Azar Gaminian, Jill Cox, Katherine French, Nalini Ranjit

**Affiliations:** a Department of Epidemiology, Center for Health Equity, The University of Texas Health Science Center at Houston, School of Public Health, 1200 Pressler St, Houston, TX 77030, USA; b Department of Health Promotion and Behavioral Sciences, Michael & Susan Dell Center for Healthy Living, The University of Texas Health Science Center at Houston School of Public Health, 1616 Guadalupe St., Austin, TX, USA; c Department of Health Promotion and Behavioral Sciences, Center for Health Equity, The University of Texas Health Science Center at Houston, School of Public Health, 1200 Pressler St, Houston, TX 77030, USA; d Brighter Bites Houston, TX, USA; e Penn State Extension Better Kid Care, 103 Innovation Blvd., Suite 214, State College, PA 16803, USA

**Keywords:** School-based, Teachers, diet, Mental health, Cardiometabolic health, Food Insecurity

## Abstract

**Background:**

We present the conceptual framework, design, and study measures of Nurturing Healthy Teachers, a quasi-experimental study to examine the short- and long-term effectiveness of the Nurturing Healthy Teachers (NHT) nutrition intervention on food insecurity, dietary behaviors, mental health and cardiometabolic health among preschool and elementary school teachers.

**Methods:**

A convenience sample of 28 elementary schools with pre-kindergarten and elementary classrooms were recruited in Houston, Texas. Nurturing Healthy Teacher intervention includes Brighter Bites, an evidence-based coordinated school health program that combines access to fresh produce and nutrition education, and Create Healthy Futures, a web-based nutrition education program that targets nutrition knowledge, self-efficacy, mindfulness, and social support to create healthy habits among teachers. The primary outcome is food insecurity. Secondary outcomes include diet quality, mental health, and cardiometabolic health. Metabolic markers and skin carotenoid levels were assessed using in-person assessments, while all other measures were obtained via questionnaire.

**Results:**

At baseline, most of the participants were female, 63 % identified as Hispanic, were highly educated, and had a mean age of 42.6 years. Overall, 50 % of teachers were classified as being obese and 20 % had high cholesterol. At baseline teachers had a mean HbA1c (%) of 5.6 %. Moderate to severe depression was experienced by 18 % of teachers and 23 % of teachers experienced moderate to severe anxiety.

**Conclusions:**

The results of this study will inform next steps towards future implementation and evaluation of teacher-focused interventions.

## Highlights

• Teachers experience food insecurity higher than national average.
• Teachers report high levels of anxiety and depression.
• Assessments demonstrate high prevalence of health conditions among teachers.
• Results warrant and inform research on teacher-focused interventions.

## 1 Introduction

More than 1.9 million elementary teachers, mostly women, provide care to over 10 million preschool and elementary school aged children in the US (National Center for Education Statistics, 2023). As adults who take care of children for a substantial part of the day, they model and cultivate healthy eating behaviors essential to children’s health and behaviors (Otten et al., 2019, Ward et al., 2017). Therefore, the health and well-being of teachers are essential to a child’s learning. However, teachers are susceptible to poor diet quality, sedentary lifestyle, and stress stemming from food insecurity (Lessard et al., 2020). Our prior studies have demonstrated that 32 % of teachers in Head Start preschools reportedly being food insecure (Mofleh et al., 2021), which is disproportionately higher than the national rates of at 12.8 % (US Department of Agriculture Economic Research Service, 2023). Furthermore, our studies have shown poor diet quality among Head Start teachers with significant inverse association between diet quality and food insecurity (Mofleh et al., 2021). Head Starts are federally funded preschools offered at no-cost to low-income children across the US. Food insecure teachers were more likely to report cost as a perceived barrier to eating fruits and vegetables as compared to their food secure counterparts (Mofleh et al., 2021). Similar findings are observed globally with recent studies in Australia reporting high levels of burnout among a third of the teachers, sub-optimal dietary habits, physical activity, and high workload being associated with these lifestyle behaviors (Corbett et al., March 22, 2023., Corbett et al., 2024).

The purpose of this study is to evaluate the impact of Nurturing Healthy Teachers, a program that combines access to fresh fruits and vegetables with nutrition education on food insecurity and health-related outcomes among teachers serving preschool (ages 3-5y) and elementary (ages 5-11y) school children in Houston, Texas. Nurturing Healthy Teachers **(NHT)**, combines strategies from two evidence-based programs - Brighter Bites and Create Healthy Futures. Brighter Bites **(BB)** is a 501c3 non-profit organization that implements an evidence-based coordinated school health program combining access to fresh produce and nutrition education proven to improve fruit and vegetable consumption and reduce food insecurity among teachers, low-income children, and their families (Sharma et al., 2016). For 16 weeks in the school year, BB teachers and families receive a weekly distribution of ∼ 20 lbs. of fresh fruits and vegetables, weekly healthy recipe tastings, and nutrition education. In prior research, over 80 % of teachers participating in BB reported improved consumption of fruits and vegetables (Sharma et al., 2016). Developed by Penn State Extension Better Kid Care (BKC), Create Healthy Futures (**CHF**) is a web-based nutrition education program with peer facilitation that targets nutrition knowledge, self-efficacy, mindfulness, and social support to create healthy habits primarily among early care and education professionals. CHF has demonstrated strong feasibility, acceptability, and improved nutrition knowledge among Head Start teachers (Cox et al. 2017). The NHT program is currently being implemented by Brighter Bites and Penn State Extension Better Kid Care in a subset of preschool and elementary schools with UTHealth Houston as the evaluation partner. The study was approved by the UTHealth Houston Committee for Protection of Human Subjects and registered on ClinicalTrials.gov before enrolling the first participant.

The primary aim of the evaluation study is to examine the effectiveness of the NHT program (intervention group) compared to CHF online course-only (comparison group) at post-intervention follow-up on improving food security prevalence among teachers. Secondary aims are to examine impacts of NHT on nutrition security, diet, mental health and well-being, and markers of metabolic health, and to evaluate the extent to which these impacts, if any, are mediated by food insecurity.

This paper presents the study design, protocol, conceptual framework, describe the study measures, and baseline data of the Nurturing Healthy Teachers study.

## 2 Methods

### 2.1 Study design

The study was designed using a cluster-based (teachers nested within schools), quasi-experimental study to be conducted over a two-year period (2022–2024). Eligible schools were assigned 1:1 into the intervention or comparison group. Random assignment was not possible because BB enrollment had already been completed for the 2022–2023 school year. Intervention schools were drawn from schools that were scheduled to receive BB during the 2022–2023 school year. While comparison schools were drawn from schools not eligible to participate in BB during the study period. due to prior exposure or had never been exposed to BB. The intervention schools were eligible to receive the BB + CHF program, while the comparison schools received CHF online modules-only. Intervention schools also received exposure to fruits and vegetables through BB over 9 months.

### 2.2 Sample size estimation

Power analyses were conducted with food insecurity prevalence as the primary outcome to estimate the number of teachers and schools to recruit. Based on prior studies in the literature, we set a target reduction for food insecurity prevalence to 15 % (from an assumed baseline of 30 %). We estimated that to detect a 50 % reduction in food insecurity prevalence with 80 % power, we would need a minimum of 320 teachers nested within 20 schools, assuming a two-tailed test and a threshold for significance set at p = 0.05.

### 2.3 Recruitment

Principals of schools from approved districts were contacted via email with a description of the study, benefits to teachers, and an offer to participate. The inclusion criteria for the schools in the evaluation included: school principal consented to participate; preschool and elementary classrooms serving children starting pre-K through grade 5; >75 % of the children in the school were participating in the free/reduced school lunch program. Teachers at the participating schools employed in some instructional capacity (classroom teacher, teaching assistant, ancillary teachers, special education teachers) were eligible to participate. All teachers meeting eligibility criteria were invited to participate. A convenience sample of 350 teachers were recruited via emails, flyers and Zoom sessions. Informed consent was obtained from participating teachers. Monetary incentives were provided to motivate attendance at the recruitment session and survey completion.

### 2.4 Intervention implementation

**Fig. 1:**
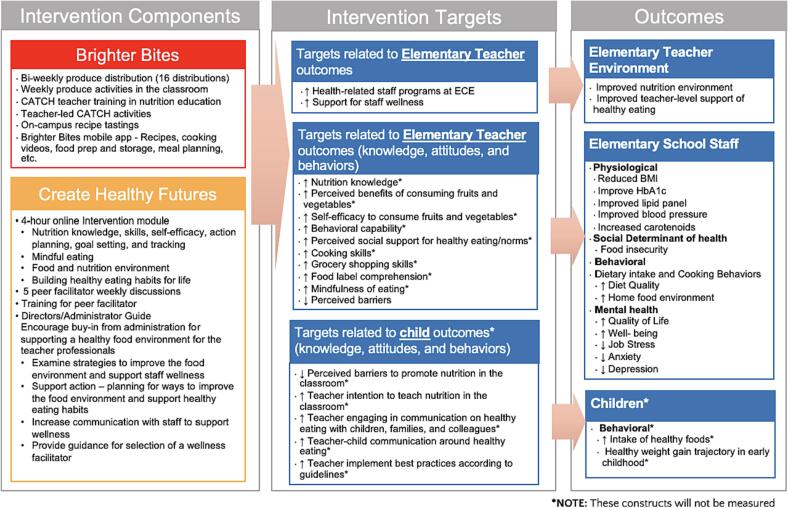
The logic model and conceptual framework for the Nurturing Healthy Teachers program.

The Nurturing Healthy Teachers program was implemented across schools in Houston, Texas between the 2022–2023 and 2023–2024 school years. The logic model presented in Fig. 1 demonstrates the program components implemented across the school year. The program components consist of the following:

1. **Create Healthy Futures Web-Based Course:** The web-based course is self-paced and focuses on the following topics: Introduction, Challenges of the food environment, Nutrition and your health, Food culture reform, and Providers' role in creating healthy futures. The course utilizes several educational methods to increase interactivity and engagement, including video footage of content experts, reflection activities, downloadable handouts, and action planning. The program is delivered on the Better Kid Care On Demand platform, an asynchronous learning management system that provides professional development training for early care and education professionals and out-of-school providers.
2. **Create Healthy Futures Peer-led Weekly Wellness Groups:** The study team, in partnership with school leadership, identified peer-facilitators to lead virtual weekly group discussions. Criteria for selection include strong communication skills, health advocate, and high regard amongst peers. Wellness facilitators include teachers participating in NHT, a school nurse, and Brighter Bites representatives. BKC trained the peer facilitators including reviewing the Facilitation Implementation Guide to provide strategies and action planning to support a healthier food environment for educators within their work setting. Each wellness facilitator led a group of up to 15 teachers and met once a week for six weeks. Wellness groups' goals were designed to encourage group members to complete a chapter of the course before each meeting, set personal goals, discuss topics in the course, and identify success stories and challenges faced by teachers in the group.
3. **Brighter Bites Produce Distribution, Education, and Activities:** Intervention schools received BB produce distributions over Spring and Summer 2023. Teachers received up to 16 distributions of fresh produce boxes each consisting of 20 lbs. of 8–12 different produce items. The BB website and mobile application provided nutrition education materials, recipe ideas, food preparation and storage ideas, tips and tools to budget and plan meals.

### 2.5 Timeline

Baseline measures were conducted in Fall 2022 (November-December) and Spring 2023 (January-April). Post-intervention measures are conducted in Fall 2023 (October-December). Long-term follow-up measures are planned for Spring 2024 (February-May). Intervention schools received BB in Spring 2023 (January-June) and Summer 2023 (June-August). All teachers received access to complete CHF online modules from January-December 2023. Facilitated CHF meetings were held from May to June 2023 for the intervention schools only.

Biometric outcome measures are conducted at baseline and post-intervention at the schools during regular school hours. Self-reported data on food security, mental health, and dietary behaviors are obtained via online surveys at baseline, post-intervention, and long-term follow-up. Process evaluation data are collected at the beginning and throughout the program.

### 2.6 Measures

#### 2.6.1 Survey measures

##### 2.6.1.1 Demographics

At baseline, teachers provided demographic information including gender, age, education level, race and ethnicity, number of children and adults in the household, marital status, length of time as a teacher, employment status, whether or not they had health insurance, and teaching position. Categories of these variables are reflected in tables.

##### 2.6.1.2 Dietary Screener

At baseline and post-intervention, dietary intake is assessed by the previously validated 2009–2010 NHANES Dietary Screener Questionnaire (DSQ), a self-administered online 26-item assessment of dietary intake over the past month (National Cancer Institute, 2015, Thompson et al., 2017). Responses to the 26 items allow calculation of several diet measures, expressed in terms of adherence to recommended daily values, including percent added sugars, fruit and vegetable consumption, whole grain, daily fiber, dairy and calcium consumption.

##### 2.6.1.3 Food security and nutrition security

At baseline, post-intervention, and long-term follow-up, food security (defined as the ability to obtain adequate food in socially acceptable ways) is assessed using the validated 6-item United States Department of Agriculture (USDA) Adult Food Security Survey Module for the last 30 days (coding details provided in Blumberg et al., 1999, Bickel et al., 2000). The food security scale distinguishes across four levels of severity of food insecurity, ranging from very low to high food security. Nutrition security is assessed using the 4-item validated Nutrition Security Screener in the last 30 days (Calloway et al., 2022), and is intended to assess factors associated with a household’s ability to obtain foods that meet their nutritional and health needs. Each of the 4 items is associated with a 5-point Likert scale, allowing a score range of 0–16.

##### 2.6.1.4 Mental health and well-being

At baseline, post-intervention, and long-term follow-up, mental health and well-being are assessed. Two measures of mental health are assessed**.** The Generalized Anxiety Disorder (GAD-7) questionnaire is a seven-item scale designed to evaluate the patient’s health status during the previous 2 weeks (Spitzer et al., 2006, Löwe et al., 2008), classifies patients using the following categories of anxiety: “severe”, “moderate”, “mild”, “minimal”. The 9-item Patient Health Questionnaire (PHQ-9), a depression module that diagnoses specific disorders using criteria from the Diagnostic and Statistical Manual of Mental Disorders (Kroenke et al., 2001), can classify patients in each of the following depression categories: “severe”, “moderately severe”, “moderate”, “mild”, “minimal”. Well-being is assessed using the World Health Organization Well-Being Index (WHO-5), a five-item self-report questionnaire that assesses subjective psychological well-being (Topp et al., 2015). Well-being categories identified by the WHO-5 include: “normal well-being”, “possible depression”, “likely depression”.

#### 2.6.2 Process evaluation measures

For Brighter Bites, the intervention group completes a process evaluation survey administered electronically at the end of spring and summer distributions on acceptability, usage, and perceived effectiveness of each of the BB program components (produce distribution, nutrition education materials, recipe tasting). BB will also provide tracking data on the number of distributions attended by participating teachers. For the BB process evaluation, the number of distributions attended by participating teachers, total number of children served by participating teachers, number of teachers that completed the program, percentage of teachers approached and attended the program, percentage of teachers participated in the program and finished, and average time for participating teachers to complete the program are all recorded by Brighter Bites and shared with UTHealth for analysis.

For Create Healthy Futures, all teachers complete a process evaluation survey administered electronically on CHF program satisfaction. For the CHF process evaluation, the number and percentage of teachers who accessed and completed the online CHF program module, the percentage who completed the program in the 6-week designated period, and the number of wellness group sessions attended are recorded by Penn State BKC and shared with UTHealth for analysis.

#### 2.6.3 Biometric measures

##### 2.6.3.1 Skin carotenoid levels

Skin carotenoid levels are assessed by the Veggie Meter® which uses reflection spectroscopy to measure skin carotenoid absorption. Veggie Meter® scores range from 0 to 800 (Ermakov and Gellermann, 2012, Ermakov et al., 2016). Three consecutive readings are done using the index finger. The Veggie Meter® is responsive to changes in dietary patterns related to fruit and vegetable consumption (Ermakov and Gellermann, 2012, Ermakov et al., 2016, Jilcott Pitts et al., 2018). The Veggie Meter® has been validated by examining the correlation between plasma carotenoids and Veggie Meter® carotenoids in a sample of racially diverse participants (Jilcott et al., 2018).

##### 2.6.3.2 Blood pressure

Blood pressure (BP) is measured with an automated BP monitor (OMRON IntelliSense Blood Pressure Monitor) by trained study staff. Three consecutive measurements are taken with a one-minute rest between each measure. The American Heart Association guidelines for blood pressure were used to classify the results: normal BP (<120/80 mmHg), elevated BP (120–129/<80 mmHg), stage 1 hypertension (130–139/80–89 mmHg), and stage 2 hypertension (≥140/90 mmHg) (Flack et al., 2020). Participants are emailed and advised by our study’s physician to consult with their primary care physician, if their blood pressure fell in any of the following categories: Elevated, Stage 1, Stage 2, or Hypertension Crisis.

##### 2.6.3.3 Glycosylated hemoglobin (HbA1c)

Blood samples are collected from each participant to measure glycosylated hemoglobin (HbA1c) levels as a measure of long-term glucose control. HbA1C levels were performed using the Siemens HbA1c DCA Vantage Analyzer. A lancing device is used to fingerstick the participants and the blood was collected into a capillary tube and placed into the analyzer. The American Diabetes Association’s Guidelines are used to classify HbA1c results: normal (4.1–5.6 %), pre-diabetic (5.7–6.4 %), diabetic (6.5 %+), and abnormally low (<4.0 %) (American Diabetes Association, 2022). Participants are emailed and advised by our study’s physician to consult with their primary care physician, if their results indicated abnormal HbA1c levels (>5.7 %).

##### 2.6.3.4 Body mass Index (BMI)

Height is self-reported by the participant at baseline. Weight is measured three consecutive times using DORAN DS6100 scale by trained study staff. Height and weight are used to compute BMI, calculated as weight in kilograms divided by height in square meters. Participants are classified into the following weight status categories based on their BMI: underweight (BMI < 18.5), normal weight (BMI 18.5–24.9), overweight (BMI 25.0–29.9), and obese (BMI ≥ 30) (Center for Disease Control and Prevention, 2022).

## 3 Results

A total of 28 schools were recruited with 16 intervention schools and 12 comparison schools. Teacher consent was obtained both online and in-person. In total, 191 teachers were consented in the intervention group and 188 in the comparison group.

### 3.1 Sample demographics

Sample demographics are summarized in Table 1. At baseline, survey data was collected from 337 participants (94.9 %). Most of the participants were female (92 %) and mean age of 42.6 years. Sixty-three percent of the teachers identified as Hispanic, while only 12 % identified as White. Participants were highly educated with 86 % having a 4-year college degree or better completed. Ninety percent of our participants had health insurance. Nearly a third of our study population were food insecure (32 %).

**Table 1:** Baseline Demographic Characteristic of Preschool and Elementary Teachers (N = 337) in Houston, Texas Stratified by Intervention Group, 2022–2023 School Year.

	Overall	Comparison Group	Intervention Group	p-value
**Age (year) (mean/std)**	42.62 ± 13.22	43.08 ± 12.83	42.16 ± 13.61	0.527
**Female (n/%)**	320 (92.22)	158 (93.49)	162 (91.01)	0.389

**Education (n/%)**				0.714
Less than high school	5 (1.45)	3 (1.79)	2 (1.12)	
High school	7 (2.02)	3 (1.79)	4 (2.25)	
Some college	32 (9.25)	12 (7.14)	20 (11.24)	
College graduate	206 (59.54)	104 (61.90)	102 (57.30)	
Post-graduate	96 (27.75)	46 (27.38)	50 (28.09)	

**Ethnicity (n/%)**				0.001*
Hispanic	201 (58.77)	82 (49.70)	119 (67.23)	
Non-Hispanic	141 (41.23)	83 (50.30)	58 (32.77)	

**Race (n/%)**				0.000*
Black	97 (28.61)	67 (41.36)	30 (16.95)	
Hispanic	179 (52.80)	71 (43.83)	108 (61.02)	
White	39 (11.50)	14 (8.64)	25 (14.12)	
Other	25 (7.07)	11 (6.18)	14(7.90)	

**Health insurance (n/%)**				0.742
Yes	320 (94.40)	156 (93.98)	164 (94.80)	
No	19 (5.60)	10 (6.02)	9 (5.20)	
*Chi-square or T-test, as appropriate.				

### 3.2 Baseline data

#### 3.2.1 Dietary measures

**Fig. 2:**
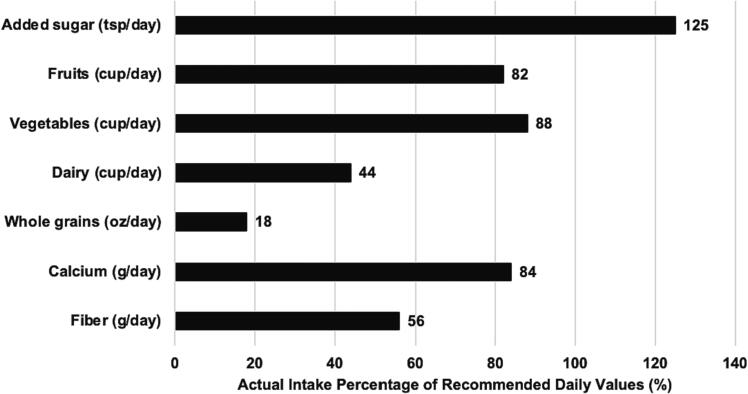
Dietary consumption as a percentage of recommended daily values of preschool and elementary teachers (N = 337) at baseline in Houston, Texas, 2022–2023 School Year.

Diet measures, assessed as actual intake percentage of recommended daily values, are presented in Fig. 2. Self-report data showed that our participants consume 125 % of added sugars (tsp/day). At baseline, fruit and vegetable consumption (cup/day) was at 82 % and 88 % of recommended daily values, respectively. Participants consumed 18 % of their recommended daily whole grain (oz/day) intake and were consuming, on average, 56 % of their recommended daily fiber (g/day). Dairy consumption (cup/day) was averaged at 44 % and calcium (g/day) at 83 % of recommended.

#### 3.2.2 Food and nutrition security

Food security was categorized into 4 categories presented in Table 2. Overall, 101 (31.96 %) of our participants were food insecure (intervention group 59 (36.88 %), comparison group 42 (26.92 %), p-value = 0.058) or 105 (34.43 %) of our participants were very low/low/marginal food secure (interventional 58 (37.66 %), comparison 47 (31.13 %), p-value = 0.67). Nutrition security was categorized into 2 categories presented in Table 2. Overall, 87 (27.19 %) of our participants were nutrition insecure too.

**Table 2:** Baseline Results for Diet, Mental Health, and Biometric Measurements of Preschool and Elementary Teachers (N = 337) in Houston, Texas Stratified by Intervention Group, 2022–2023 School Year.

	Overall	Comparison Group	Intervention Group	p-value
**Dietary Habits**				
Fiber (g/day)	14.06 ± 2.09	14.05 ± 2.22	14.07 ± 1.97	0.946
Calcium (g/day)	830.14 ± 125.27	821.54 ± 134.45	838.73 ± 115.13	0.216
Whole grains (oz/day)	0.58 ± 0.23	0.60 ± 0.261	0.56 ± 0.19	0.178
Added sugar (tsp/day)	14.89 ± 5.11	15.25 ± 5.99	14.53 ± 4.03	0.200
Dairy (cup/day)	1.31 ± 0.36	1.27 ± 0.37	1.34 ± 0.33	0.081
Fruit and vegetables, including legumes and French fries (cup/day)	2.24 ± 0.53	2.24 ± 0.55	2.23 ± 0.52	0.911
Vegetables including legumes and French fries (cup/day)	1.43 ± 0.27	1.43 ± 0.26	1.43 ± 0.27	0.994
Fruits and vegetables, including legumes and excluding French fries (cup/day)	2.13 ± 0.55	2.14 ± 0.57	2.12 ± 0.54	0.703
Vegetables, including legumes and excluding French fries (cup/day)	1.32 ± 0.30	1.32 ± 0.29	1.32 ± 0.30	0.988
Fruits (cup/day)	0.82 ± 0.34	0.83 ± 0.36	0.81 ± 0.32	0.572
Added sugars from sugar-sweetened beverages (tsp/day)	6.83 ± 4.86	7.11 ± 5.74	6.55 ± 3.80	0.294

**Food and Nutrition Security**				
**Food Secure 6-item (n/%)**				0.67
Very low food security	17 (5.57)	8 (5.30)	9 (5.84)	
Low food security	46 (15.08)	21 (13.91)	25 (16.23)	
Marginal food security	42 (13.77)	18 (11.92)	24 (15.58)	
Food secure	200 (65.57)	104 (68.87)	96 (62.34)	

**Nutrition Secure (n/%)**				0.69
Insecure	87 (27.19)	44 (28.21)	43 (26.22)	
Secure	250 (72.81)	127 (71.79)	123 (73.78)	

**Mental Health**				
**Depression (n/%)**				0.75
Severe	7 (2.13)	4 (2.42)	3 (1.84)	
Moderately severe	16 (4.88)	6 (3.64)	10 (6.13)	
Moderate	37 (11.28)	20 (12.12)	17 (10.43)	
Mid	85 (25.91)	40 (24.24)	45 (27.61)	
Minimal	183 (55.79)	95 (57.58)	88 (53.99)	

**Well-being (n/%)**				0.17
Normal well-being	186 (56.53)	96 (59.26)	90 (53.89)	
Possible depression	84 (25.53)	34 (20.99)	50 (29.94)	
Likely depression	59 (17.93)	32 (19.75)	27 (16.17)	

**Anxiety (n/%)**				0.34
Severe	31 (9.51)	13 (8.02)	18 (10.98)	
Moderate	44 (13.50)	27 (16.67)	17 (10.37)	
Mild	101 (30.98)	48 (29.63)	53 (32.32)	
Minimal	150 (46.01)	74 (45.68)	76 (46.34)	

**Biometrics Measurements**				
**Veggie Meter (carotenoid levels)**	224.32 ± 81.50	226.97 ± 77.70	222.66 ± 85.30	0.70
**Systolic Blood Pressure (mmHg)**	117.58 ± 15.39	117.28 ± 15.55	117.87 ± 15.27	0.72
**Diastolic Blood Pressure (mmHg)**	75.05 ± 10.82	74.22 ± 10.90	75.88 ± 10.70	0.15
**Hypertension (n/%)**				0.34
Yes	44 (12.46)	19 (10.80)	25 (14.12)	
No	309 (87.54)	157 (89.20)	152 (85.88)	
**HbA1c (%)**	5.63 ± 0.94	5.62 ± 0.91	5.64 ± 0.98	0.84
**HbA1c category (n/%)**				0.78
diabetic	28 (7.95)	13 (7.39 %)	15 (8.52)	
pre-diabetic	78 (22.16)	37 (21.02)	41 (23.30)	
normal	246 (69.89)	126 (71.59)	120 (68.18)	
**Weight (kg)**	84.96 ± 24.61	85.58 ± 25.54	84.34 ± 23.70	0.64
**BMI (continuous)**	31.73 ± 8.27	32 ± 8.49	31.46 ± 8.06	0.55
**BMI category (n/%)**				0.73
Obesity (BMI>=30.0)	169 (50.15)	84 (50.91)	85 (49.42)	
Overweight	97 (28.78)	44 (26.67)	53 (30.81)	
Healthy weight	68 (20.18)	36 (21.82)	32 (18.60)	
Underweight	3 (0.89)	1 (0.61)	2 (1.16)	
*Chi-square or T-test, as appropriate.				

#### 3.2.3 Mental health and well-being

Mental health and well-being of teachers was assessed by self-reports using the GAD-7, PHQ-9, and WHO-15 Well Being Index and CDC HRQOL– 4. Nearly a quarter of teachers (23 %) were classified as having moderate to severe anxiety on the GAD-7 measure, while 18 % were assessed as having moderate to severe depression on the PHQ-9. Notably, only about half of the teachers fell in the category of minimal depression and anxiety, and just 43 % of the study population was categorized as having a normal well-being, suggesting that a substantial proportion of teachers suffer suboptimal mental health.

#### 3.2.4 Self-Report chronic Disease prevalence

**Fig. 3:**
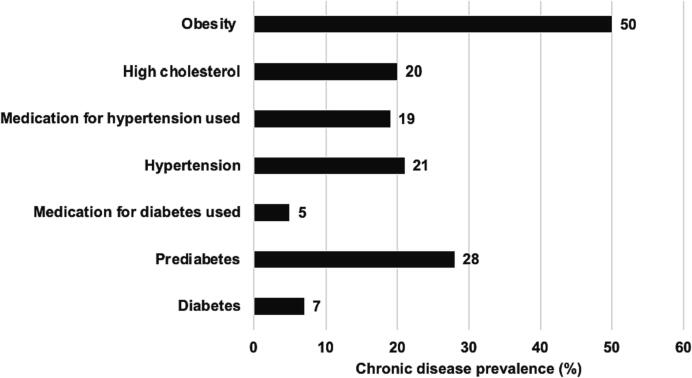
Self-report diagnosis of chronic conditions of preschool and elementary teachers (N = 337) at baseline in Houston, Texas, 2022–2023 School Year.

Teachers self-reported height, which was used to compute BMI and weight status, and diagnosis of high cholesterol, diabetes, and hypertension are presented in Fig. 3. Overall, fifty percent of our participants were categorized as obese. High cholesterol was reported in 20 % of our participants. There were 28 % who self-reported as prediabetic and 7 % self-reported as having diabetes. Five percent reported that they took medication for their diabetes. Twenty-one percent self-reported to have hypertension and 19 % self-reported to take medication for their hypertension.

#### 3.2.5 Biometric measurements

At baseline, biometric measurements were collected for 353 teachers. Most biometric measures were comparable across intervention and comparison groups (Table 2). Veggie Meter ® scores were similar in both groups (intervention Mean = 222.66, SD=±85.31, comparison Mean = 225.97, SD=±77.72, p-value 0.704), as were systolic and diastolic blood pressure levels. Approximately 12 % of teachers, across both arms, were hypertensive, using American Heart Association guidelines (18). The intervention group had a higher percentage of those with hypertension (14.12 %) compared to the comparison group (n = 19, 10.80 %), but differences were not significant. At baseline, teachers had a mean HbA1c (%) of 5.6 %, normal glucose level and mean BMI of 31.7. Mean HbA1c (%) and BMI were also similar among both the intervention and comparison group as seen in Table 2. About half of the teachers in this study were classified as obese, and another 28 % as overweight; again, these prevalences were comparable across study arms.

## 4 Discussion

Our study is one of the first to rigorously assess the impact of a multi-level nutrition intervention consisting of access to fresh produce plus nutrition education on physical and mental health outcomes, diet quality and food security among teachers predominantly employed in schools serving low-income children and their families. Additionally, we will understand and assess the factors informing implementation success of the program. In recent years, there has been increasing popularity of programs to reduce food insecurity and improve access to healthy food in food secure populations using schools, clinics and other trusted venues and partners as implementing sites for these efforts (Norris et al., 2023). However, the predictors of implementation success, and the effectiveness of these programs across diverse populations and settings needs further research. Understanding these factors can impact scalability and sustainability of programs.

Rates of food insecurity were > 30 % which is over two times the national average of 12.8 % (Rabbitt et al., 2022) with concurrently less than two cups of fruits and vegetables consumed at baseline. These results are congruent with our prior studies that have demonstrated higher rates of food insecurity and concurrent poor diet quality among teachers (Mofleh et al., 2021). Moreover, while there is a plethora of studies on healthy nutrition interventions among children in preschool and school settings (Collado-Soler et al., 2023, Cotton et al., 2020), few studies have implemented teacher-focused interventions and evaluated their impact on physical and mental health-related outcomes (Mofleh et al., 2022). Many of these child-focused interventions rely on teachers to act as agents of intervention implementation and success. Thus, it is imperative to ensure that teachers have the ability, knowledge, and resources to engage in these healthy behaviors themselves. Our study aims to fill these gaps in the current literature by implementing a multi-level teacher-focused nutrition intervention and assessing its impact on behavioral, social, environmental and health outcomes.

Furthermore, our baseline biometric data demonstrates high prevalence of diabetes (8 %), hypertension (12.5 %), and obesity (50 %) among participating school teachers. Concurrently, 43 % of our participating teachers reported less than normal well-being, 23 % reported moderate-to-severe anxiety, and 18 % reported moderate-to-severe depression. These results concur with prior studies that have also demonstrated high levels of stress and burnout among teachers which can impact the quality of education imparted in the classrooms (Li et al., 2022). While there could be a selection bias associated with the teachers enrolled in our study such that those who are “sicker” may be more likely to participate to address their health conditions, these results warrant immediate attention. Additionally, school-based research has shown that teachers are largely responsible for implementing health education curriculum in the schools (Collado-Soler et al., 2023). Some studies have reported that teachers bear the responsibility of teaching health education to students but may utilize substandard resources that lack alignment with the curriculum and may fail to incorporate effective teaching methodologies aimed at improving student outcomes (Peralta et al., 2016). Recent scoping review of the literature has shown median prevalence of stress, burnout, anxiety, and depression among teachers to respectively be 67.0 %, 60.9 %, 39.6 %, and 14.0 % with a call to action to implement interventions to address these issues (Agyapong et al., 2022).

Our intervention components are theoretically grounded in the Social Cognitive Theory and Theory of Planned Behavior (Bandura, 2001, Ajzen, 1991). Furthermore, we are leveraging on two evidence-based strategies – Brighter Bites, and Create Healthy Futures, to improve teacher health (Mofleh et al., 2022, Sharma et al., 2015, Sharma et al., 2017). Our robust evaluation framework includes a fully powered sample size, a comparison group, and objective measures of fruit and vegetable consumption, and biometrics. Our implementation outcomes are critical to assess dose–response relationships in the outcome analysis. We will also use these data to inform implementation of such programs in school/preschool settings to reach teachers.

Finally, our study seeks to share the school workflows and environmental supports needed to support implementation of programs such as Brighter Bites and Create Healthy Futures, and support training of teachers in nutrition education.

### 4.0.1 Strengths and Limitations

Our study has several strengths. We have a large number of “clusters” at the school level which lends power to the overall sample size. Our study is fully powered for food insecurity as a primary outcome and has a usual care wait list comparison group. This allows us to compare outcomes across both groups. The use of objective measures of clinical indicators and biomarkers as well as fruit and vegetable intake using the Veggie Meter is a strength. We are leveraging existing partnerships with school systems, and assets of evidence-based programs, Brighter Bites and Create Healthy Futures. If found to be effective, a platform already exists for dissemination. Finally, we are assessing short- and longer-term impact of the program. These strengths notwithstanding, our study has limitations. Self-reported data on surveys has social desirability bias. Given that Brighter Bites recruits their school one school year ahead of implementation, we could not do random assignment of schools to intervention or comparison group. This is a convenience sample of schools and as such could be subject to selection bias. Additionally, sampling was not evenly distributed across intervention and control groups. However, our baseline data shows no significant difference between the two groups for the outcomes of interest.

## 5 Conclusions

The results of this study will inform next steps towards future implementation and evaluation of teacher-focused interventions. Notably, this study was made possible through partnerships with school districts, multiple agencies that have a national reach for programming, and evaluation through UTHealth Houston. Each of these groups collectively brings the experience and resources needed to successfully address the needs of at-risk teacher population.

## Funding Source

Funding for this study was provided by the Vitamix Foundation. The study was also supported by the UTHealth Houston School of Public Health Center for Health Equity.

**Ethical considerations & disclosure(s)**.

This project was approved by the University of Texas Health Science Center, Committee for Protection of Human Subjects (HSC-SPH-22–0276). Informed consent was obtained from participants prior to participating in this study. The clinical trial was registered on ClinicalTrials.gov (NCT05542537) on September 2022, prior to enrollment of the first participant.

## CRediT authorship contribution statement

**Shreela V. Sharma:** Writing – review & editing, Writing – original draft, Validation, Supervision, Resources, Project administration, Methodology, Investigation, Funding acquisition, Data curation, Conceptualization. **Mackenzie Senn:** Writing – review & editing, Writing – original draft, Validation, Supervision, Project administration, Investigation, Data curation. **Angela Zieba:** Writing – review & editing, Supervision, Investigation, Data curation. **Miao Tang:** Writing – review & editing, Formal analysis, Data curation, Conceptualization. **Ru-Jye Chuang:** Writing – review & editing, Supervision, Investigation. **Courtney Byrd-Williams:** Writing – review & editing, Conceptualization. **Mike Pomeroy:** Writing – review & editing, Supervision, Resources, Project administration, Methodology, Conceptualization. **Azar Gaminian:** Writing – review & editing, Supervision, Project administration, Methodology, Investigation. **Jill Cox:** Writing – review & editing, Supervision, Project administration, Methodology, Investigation, Funding acquisition, Conceptualization. **Katherine French:** Writing – review & editing, Supervision, Project administration, Investigation, Conceptualization. **Nalini Ranjit:** Writing – review & editing, Writing – original draft, Supervision, Methodology, Funding acquisition, Formal analysis, Data curation, Conceptualization.

## Declaration of competing interest

The authors declare the following financial interests/personal relationships which may be considered as potential competing interests: Dr. Shreela Sharma serves on the board of directors of Brighter Bites 501c3 non-profit organization. This is an unpaid board position. No other authors report any conflicts of interest..

## Data Availability

Data will be made available on request.
